# Lesion locations are associated with cognitive impairment after ischemic stroke in young adults

**DOI:** 10.1016/j.nicl.2025.103930

**Published:** 2025-12-17

**Authors:** Mijntje M.I. Schellekens, Hao Li, Maartje Wijnands, Anastasia Papounidou, Esther M. Boot, Jamie I. Verhoeven, Merel S. Ekker, Mayte E. van Alebeek, Paul J.A.M. Brouwers, Renate M. Arntz, Gert W. van Dijk, Rob A.R. Gons, Inge W.M. van Uden, Tom den Heijer, Julia H. van Tuijl, Karlijn F. de Laat, Anouk G.W. van Norden, Sarah E. Vermeer, Marian S.G. van Zagten, Robert J. van Oostenbrugge, Marieke J.H. Wermer, Paul J. Nederkoorn, Frank G. van Rooij, Ido R. van den Wijngaard, Paul L.M. de Kort, Vitória Piai, Frank-Erik de Leeuw, Roy P.C. Kessels, Anil M. Tuladhar

**Affiliations:** a Department of Neurology, Radboud University Medical Centre, Donders Institute for Brain, Cognition and Behaviour, Nijmegen, the Netherlands; b Department of Neurology, Gelre Hospitals, Apeldoorn, the Netherlands; c Department of Neurology, Medisch Spectrum Twente, Enschede, the Netherlands; d Department of Neurology, Canisius-Wilhelmina Hospital, Nijmegen, the Netherlands; e Department of Neurology, Catharina Hospital, Eindhoven, the Netherlands; f Department of Neurology, Franciscus Gasthuis & Vlietland, Rotterdam, the Netherlands; g Department of Neurology, Elisabeth-TweeSteden Hospital, Tilburg, the Netherlands; h Department of Neurology, Haga Hospital, Den Haag, Netherlands; i Department of Neurology, Amphia Hospital, Breda, the Netherlands; j Department of Neurology, Rijnstate Hospital, Arnhem, the Netherlands; k Department of Neurology, Jeroen Bosch Hospital, ’s-Hertogenbosch, the Netherlands; l Department of Neurology, School for Mental Health and Neuroscience (MHeNs), Maastricht University Medical Centre, Maastricht, the Netherlands; m Department of Neurology, Leiden University Medical Centre, Leiden, the Netherlands; n Department of Neurology, University Medical Centre Groningen, Groningen, the Netherlands; o Department of Neurology, Amsterdam University Medical Centre, location AMC, Amsterdam, the Netherlands; p Department of Neurology, Medical Centre Leeuwarden, Leeuwarden, the Netherlands; q Department of Neurology, Haaglanden Medical Centre, Den Haag, the Netherlands; r Donders Institute for Brain, Cognition and Behaviour, Centre for Cognition, Radboud University, Nijmegen, the Netherlands; s Vincent van Gogh Institute for Psychiatry, Venray, the Netherlands; t Radboudumc Alzheimer Centre, Radboud University Medical Centre, Nijmegen, the Netherlands

**Keywords:** Lesion-symptom mapping, Stroke, Cognitive impairment

## Abstract

**Introduction:**

Stroke location is an important determinant of post-stroke cognitive impairment (PSCI). In young adults, a comprehensive map of lesion patterns and their relations to PSCI is lacking. This study aims to identify lesion locations associated with poorer cognitive performance in patients with stroke at a young age.

**Methods:**

We conducted a multicenter prospective cohort study between 2013 and 2021, enrolling patients aged 18–49 years with first-ever ischemic stroke and a visible stroke lesion on MRI. Cognitive assessments were performed within six months post-stroke, covering seven domains. We categorized patients as having no/mild or major vascular cognitive disorder (VCD), defined as a Z-score < -2.0 in one or more domains. We assessed aphasia by the NIHSS language subscale. We performed multivariate lesion-symptom mapping to identify lesion locations associated with major VCD, poorer cognitive performance in each domain, and aphasia.

**Results:**

Among 522 patients (median age 44.3 years [IQR 37.7–41.5]; 257 [49.2 %] women), 168 (32.2 %) had major VCD. Lesions in both hemispheres and cerebellar regions were associated with presence of a major VCD, and lower performance in episodic memory, processing speed, executive functioning, language, and attention and working memory. Aphasia had the strongest relationship with left fronto-temporo-parietal regions, while the left angular gyrus was the region most associated with major VCD.

**Discussion:**

We show that lesion locations associated with poorer cognitive performance in young stroke patients are widely distributed, including cerebellar regions. This study showcases the complexity in the relationships between affected brain regions and cognitive symptoms, explaining the variability in post-stroke cognitive outcome.

## Highlights

• Stroke lesions associated with poorer cognitive performance are widely distributed.
• Lesions in the cerebellum are also associated with poorer cognitive performance.
• Lesion distribution may explain the variability in cognitive deficits after stroke.

## 1 Introduction

Each year, more than two million young adults (18–50 years) suffer an ischemic stroke worldwide (Béjot et al., 2016, Global, regional, and national burden of stroke, 2016). Stroke location is, in addition to age, education level, vascular risk factors, stroke severity and lesion volume, an important determinant of post-stroke outcome (Weaver et al., 2021, Pendlebury and Rothwell, 2019). Recent studies using lesion-symptom mapping, investigating the relationship between structural damage and behavioral deficits, have provided further evidence for the role of strategic infarct location in post-stroke cognitive impairment (PSCI) (Weaver et al., 2021, Munsch et al., 2016, Zhao et al., 2018, Bowren et al., 2022). However, these studies involved older patients, who often have a history of stroke in addition to the index event and age-related neurodegenerative pathology. Additionally, not all of these studies used extensive cognitive assessments and, consequently, a comprehensive lesion-symptom map is still lacking in young adults. The relationship between stroke location and post-stroke cognitive performance may differ from stroke patients > 50 years, as young adults are less likely to have coexisting neurovascular changes, such as cerebral small vessel disease or neurodegenerative disorders. This suggests that lesion-symptom mapping results are less affected by such confounding factors in this group. Clinical MRI scans in the acute phase may be used to predict cognitive outcomes in the first months after stroke, which could be valuable for early information provision and rehabilitation planning.

Multivariate lesion analysis of structural imaging data is a lesion-symptom mapping (LSM) method (Smith et al., 2013, Mah et al., 2014, Zhang et al., 2014) that identifies the entire lesion-symptom association pattern simultaneously, rather than assessing the brain-behavior relation at each voxel separately, as in traditional voxel-based lesion-symptom mapping (Bates et al., 2003). These multivariate techniques are better at identifying multiple critical foci within distributed networks, as well as diffuse brain-behavior relationships, such as complex cognitive functions, compared to traditional voxel-based lesion-symptom mapping (Pustina and Mirman, 2022).

The objective of this study is to evaluate lesion locations associated with PSCI in young adults. For this purpose, we performed a multivariate LSM study in a large cohort of patients with stroke at a young age and investigated whether lesion locations are associated with poorer cognitive performance.

## 2 Methods

### 2.1 Patients and study design

This study is a part of the *‘Observational Dutch Young Symptomatic StrokE studY’ (ODYSSEY)*, a multicenter prospective cohort study investigating risk factors and prognosis of stroke in young adults (Schellekens et al., 2023, Arntz et al., 2014). The participating centers of the ODDYSEY study are listed in the Supplementary Methods section. For the current study, we included patients aged 18–49 years with a first-ever ischemic stroke with radiological evidence of cerebral ischemia on MRI. Inclusions took place between May 2013 and February 2021. Exclusion criteria included a history of stroke, retinal infarction, and cerebral venous sinus thrombosis. Further details on data collection procedures have been published previously (Arntz et al., 2014).

### 2.2 Standard protocol approvals, registrations, and patient consents

The Medical Review Ethics Committee region Arnhem-Nijmegen (NL41531.091.12) approved the study. We obtained written informed consent from all participants. If patients were unable to provide informed consent themselves, this was obtained from their legal representative.

### 2.3 Cognitive assessment

Patients underwent an extensive neuropsychological assessment (at a median time point slightly less than three months after stroke). Neuropsychological assessments were conducted by trained research assistants and specialized nurses across centers, following the same standardized training protocol. The seven most relevant cognitive domains were assessed: (i) Episodic memory, (ii) Processing speed, (iii) Visuoconstruction, (iv) Executive functioning, (v) Visual neglect, (vi) Attention and working memory, and (vii) Language. Raw test scores were converted into Z-scores per test for each participant, adjusted for individual’s age, sex and/or education level, using normative data. Further details regarding the collection and processing of cognitive data can be found in the Supplementary Methods section and elsewhere (Schellekens et al., 2023, Schellekens et al., 2023).

Cognitive impairment on a domain was defined as a composite Z-score of < -1.5. We used the criteria for vascular cognitive disorder (VCD) as proposed by the International Society for Vascular Behavioral and Cognitive Disorders (VASCOG) (Sachdev et al., 2014). We defined major VCD as a composite Z-score of < -2.0, in one or more cognitive domains (reflecting 2.3 % of the normal population). All remaining patients were classified as no/mild VCD.

### 2.4 Neuroimaging data acquisition

Structural MRI scans were performed in a clinical setting on 1.5 T or 3 T scanners. The MRI scanners and their scanning parameters are listed in the Supplementary Methods section. The imaging protocol included at least a clinical diffusion-weighted imaging (DWI) and a fluid attenuated inversion recovery (FLAIR) scan.

## 3 Neuroimaging data processing

### 3.1 Lesion segmentation

All stroke lesions were segmented using the semi-automated region-growing method with active contour refinement as available within ITK-SNAP ITK-SNAP (Yushkevich et al., 2016, Yushkevich et al., 2006). Lesions were segmented on DWI (n = 468) when identified approximately within two weeks from the index event; on FLAIR (n = 52) when identified approximately after two weeks, or, if unavailable, on T1 (n = 1) or T2 (n = 1) sequences. Each lesion mask was visually inspected and manually adjusted if necessary. All lesion masks were reviewed for consistency by a single trained researcher.

### 3.2 Spatial normalization

Using Advanced Normalization Tools (ANTs, v 2.1.0) (Tustison et al., 2010), we registered brain images and corresponding lesion mask to the Montreal Neurological Institute (MNI) 152 ICBM 2009c Nonlinear Symmetric template, a standard space in neuroimaging. To improve the registration, the anatomical images were bias corrected, denoised and skull-stripped. The registration was performed by moving the template to the patients’ image and then using the inverse transformation to bring the patients’ image into standard space. If the registration was not possible with ANTs, we used elastix (Klein et al., 2010), a software program for intensity-based medical image registration, to directly register the skull-stripped images to the MNI 152 ICBM 2009c Nonlinear Symmetric space. Following normalization, all images and lesion masks were visually inspected for quality and alignment by a single researcher. In three patients, the registration of the lesion to the template was unsuccessful for both methods due to anatomical (n = 1) or technical (n = 2) reasons, leading to their exclusion from the analysis. We calculated normalized lesion volumes using FSLstats (Jenkinson et al., 2012).

### 3.3 Other measurements

We scored the level of education of the patients with a Dutch scoring system comprising seven categories (Verhage, 1964) that approximately align with the UNESCO international classification of education levels (UNESCO, 2011). At the time of the baseline cognitive assessment, we evaluated symptoms of depression using the Mini International Neuropsychiatric Interview (MINI) (Sheehan et al., 1998) and measured fatigue using the subscale Subjective Fatigue of the revised Checklist Individual Strength (CIS-20R) (Vercoulen et al., 1994). We determined functional outcome at the time of the baseline cognitive assessment using the Barthel Index (Mahoney and Barthel, 1965) and modified Rankin Scale (mRS) (van Swieten et al., 1988). To quantify stroke severity at admission and discharge, we used the National Institutes of Health Stroke Scale (NIHSS) (Brott et al., 1989), applying a validated retrospective scoring method when necessary (Kasner et al., 1999, Williams et al., 2000). Finally, we classified stroke etiology according to the modified Trial of ORG 10172 in Acute Stroke Treatment (TOAST) (Hennerici and Investigators, 2009). Compared to the original TOAST classification, the classification used in the present study includes an additional category, ‘likely atherothrombotic’, as young patients may not always present with significant cervical artery stenosis but may still have clear cardiovascular risk factors. The most common rare causes of stroke in the ODYSSEY study were carotid or vertebral artery dissection, antiphospholipid syndrome, and illicit drug use. Detailed information on the etiology of patients participating in the ODYSSEY study can be found elsewhere (Ekker et al., 2023).

### 3.4 Statistical analysis

We compared baseline characteristics between patients with no/mild VCD versus major VCD, using independent *t*-test or Mann-Whitney *U* test for continuous variables and Pearson’s Chi-squared test for categorial variables. To examine whether lesion volume was associated with cognitive performance within VCD severity groups, we calculated Spearman’s rank correlation coefficients (ρ) between normalized lesion volume and domain-specific cognitive Z-scores. We adjusted p-values using the Benjamini–Hochberg procedure to control the false discovery rate (FDR).

#### 3.4.1 Multivariate lesion-symptom mapping

We performed multivariate LSM using the spare canonical correlation analysis (SCCAN) as implemented in the LESYMAP package (version: 0.0.0.9222) in R (Team RC). In brief, SCCAN maps the associations between brain regions and cognitive deficits by identifying sets of voxels that collectively explain variance in cognitive performance scores (multivariate method). We excluded voxels with minimal coverage (fewer than five lesions), which is a standard approach (Bowren et al., 2022). All vascular territories were assessed. LESYMAP runs internal 4-fold cross-validation procedures to find the best sparseness. Sparseness reflects the proportion of voxels contributing to the lesion-symptom solution: lower absolute values indicate a sparser (focal) pattern, whereas higher absolute values indicate a less sparse (distributed) pattern. For interpretation in this study, only the magnitude of sparseness is relevant. To find the best sparseness, 75 % of the patients is used to identify voxel weights and 25 % is used to predict the cognitive scores with those weights. The allocation of patients into these different subgroups is performed internally and automatically by LESYMAP, without consideration of clinical or demographic factors. Once the best sparseness value is found, a final SCCAN is run on all patients usings this optimal sparseness value. After sparseness estimation, we applied a cluster-size threshold. Clusters smaller than 150 voxels were excluded, following the default setting in the LESYMAP package. Statistical significance was assessed with correction for multiple comparisons via the False Discovery Rate (FDR) method, as implemented by default in LESYMAP. We performed separate multivariate LSMs for the presence of a major VCD, Z-score of each cognitive domain, and aphasia assessed by the NIHSS language subscale at discharge. If scores were missing at random, we performed mean imputation.

To identify peak regions of interest (ROIs) with the strongest associations with the cognitive outcomes from the lesion-symptom map, a cluster tool in FSL was used. All lesion-symptom maps were spatially normalized to the MNI152 T1-weighted 1 mm template provided by FSL. We based anatomical locations of the lesions on the Harvard-Oxford cortical and subcortical structural atlas (Makris et al., 2006, Frazier et al., 2005, Desikan et al., 2006, Goldstein et al., 2007), and the JHU ICBM white-matter atlas (Mori et al., 2005, Wakana et al., 2007, Hua et al., 2008), available in FSL. We identified the structures to which the center of mass of each cluster predominantly belongs. If the center of mass was located in unclassified white matter, we additionally reported, when possible, another (sub)cortical structure or white matter tract to which the center of mass belongs. Intensity values for each cluster ranged from −1 to 1, with −1 and 1 indicating the most robust relationship between the region and the cognitive score. We report only regions with poorer cognitive performance, as it seems unlikely that stroke lesions directly improve cognition. To facilitate interpretability, we inverted the intensity values of the cognitive domain scores, as lower Z-scores indicate poorer cognitive performance, to ensure that the intensity values were positive.

#### 3.4.2 *Sensitivity* analyses

We performed sensitivity analyses to correct for lesion volume using two strategies: (1) the internal LESYMAP option and (2) the residual-based adjustment approach. Because the LESYMAP package does not support direct inclusion of other covariates than lesion volume, we performed a preprocessing step to adjust cognitive scores. Specifically, we fitted separate linear regression models for each cognitive domain using either time interval between MRI and cognitive assessment, depression status, or lesion volume as predictors. The residuals from these models, representing cognitive performance adjusted for these factors, were then used as input for the LESYMAP analyses. Furthermore, we conducted a sensitivity analysis restricted to patients who underwent cognitive assessment within the first three months, to examine the potential influence of assessment timing. Data on fatigue were missing for 87 (16.7 %) patients and were not missing at random, as patients without a fatigue score more often had a major VCD (p = 0.032). Therefore, fatigue was not imputed and was not included as a covariate.

## 4 Results

**Fig. 1:**
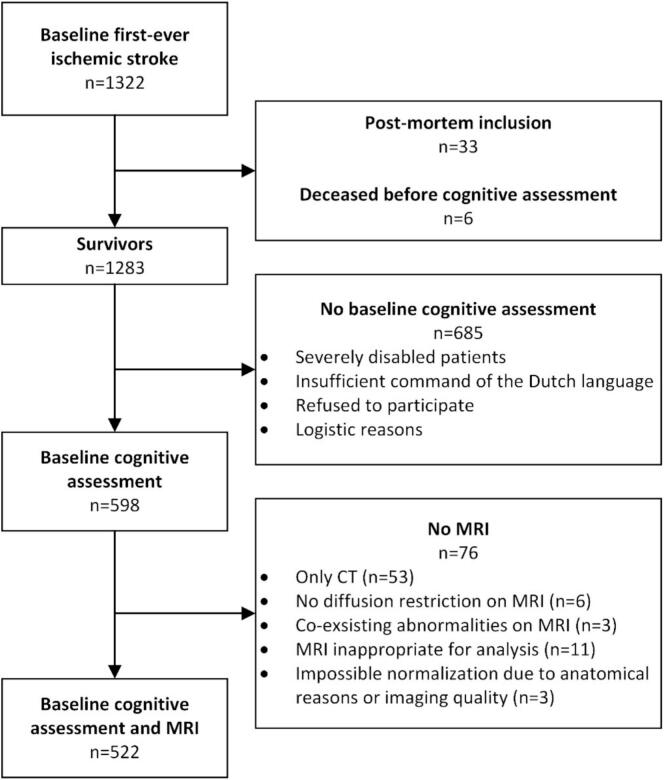
Flowchart of the study population.

Of the 1,322 patients included in the ODYSSEY study with an ischemic stroke, 522 ischemic stroke patients were included in this analysis, see Fig. 1 for a flowchart of the study population. Baseline and imaging characteristics, stratified by major or no/mild VCD, are described in Table 1.

**Table 1:** Baseline characteristics.

	**Patients**			
	**All patients** **(n = 522)**	**No/mild VCD** **(n = 354)**	**Major VCD** **(n = 168)**	**p-value**
Median age, years (IQR)	44.3 (37.7–41.5)	44.3 (38.4–47.1)	44.2 (36.4–47.8)	0.732
Men, n (%)	265 (50.8)	191 (54.0)	74 (44.4)	0.043
Median time to cognitive assessment, days (IQR)	83 (56–119)	85 (55–121)	76 (57–113)	0.262
Median education level (IQR)	5 (5–5)	5 (5–6)	5 (5–5)	<0.001
Median NIHSS score at admission (IQR)	2 (1–4)	2 (1–4)	3 (1–5)	<0.001
Median NIHSS score at discharge (IQR)	1 (0–2)	1 (0–2)	1 (0–2)	0.002
Median Barthel Index at baseline (IQR)	100 (100–100)	100 (100–100)	100 (100–100)	<0.001
Good outcome (BI ≥ 85), n (%)	488 (97.2)	336 (97.7)	152 (96.2)	
Median mRS (IQR)	1 (1–2)	1 (1–2)	1 (1–2)	0.005
Good outcome (mRS 0–1), n (%)	337 (66.7)	241 (69.7)	96 (60.4)	
Symptoms of depression present, n (%)	49 (9.6)	27 (7.7)	22 (13.9)	0.042
Mean CIS-20R − fatigue severity (SD)	32.9 (12.0)	32.0 (11.7)	35.2 (12.2)	0.011
No/mild fatigue < 36, n (%)	251 (57.7)	183 (60.2)	68 (51.9)	0.134
Severe fatigue ≥ 36, n (%)	184 (42.3)	121 (39.8)	63 (48.1)	
Median time to MRI, days (IQR)	2 (1–5)	3 (1–5)	2 (1–5)	0.620
Median normalized lesion volume, mL (IQR)	2.3 (0.7–13.3)	2.0 (0.6–9.4)	4.2 (0.8–24.5)	0.003
Lesion location, n (%)				0.058
Left supratentorial	178 (34.1)	113 (31.9)	65 (38.7)	
Right supratentorial	200 (38.3)	137 (38.7)	63 (37.5)	
Bilateral supratentorial	28 (5.4)	15 (4.2)	13 (7.7)	
Infratentorial	78 (14.9)	63 (17.8)	15 (8.9)	
Supratentorial (unilateral) and infratentorial	19 (3.6)	14 (4.0)	5 (3.0)	
Supratentorial (bilateral) and infratentorial	19 (3.6)	12 (3.4)	7 (4.2)	
TOAST, n (%)				0.379
Atherothrombotic	18 (3.4)	8 (2.3)	10 (6.0)	
Likely atherothrombotic	62 (11.8)	42 (11.8)	20 (11.9)	
Small vessel disease	80 (15.3)	53 (15.0)	27 (16.1)	
Cardioembolic	86 (16.5)	64 (18.1)	22 (13.1)	
Rare causes	102 (19.5)	68 (19.2)	34 (20.2)	
Multiple causes	31 (5.9)	21 (5.9)	10 (6.0)	
Cryptogenic	143 (27.4)	98 (27.7)	45 (26.7)	

VCD: Vascular Cognitive Disorder; IQR: interquartile range; NIHSS: National Institutes of Health Stroke Scale; BI: Barthel Index; mRS: modified Rankin Scale; MINI: Mini International Neuropsychiatric Interview; CIS-20R: Checklist Individual Strength; TOAST: Trial of ORG 10172 in Acute Stroke Treatment.

Education category 5, i.e. middle school / secondary vocational training.

Missing data: NIHSS ad admission 2 (0.4%); NIHSS at discharge 3 (0.6%); Barthel Index 20 (3.8%); MINI symptoms of depression 14 (2.7%); CIS-20R-fatigue 87 (16.7%).

Median age of patients at stroke onset was 44.3 years (IQR 37.7–41.5), 257 (49.2 %) were woman, and median NIHSS score at admission was 2 (IQR 1–4). Median time from index event to MRI was 2 days (IQR 1–5) and median normalized lesion volume was 2.3 mL (IQR 0.7–13.3). Median time from index event to cognitive assessment was 83 days (IQR 56–119). The distribution of patients assessed per month after stroke in presented in Supplementary Fig. 1. Patients with a major VCD were more frequently women (p = 0.043), had a lower education level (p < 0.001), a higher NIHSS score at admission (p < 0.001), and at discharge (p = 0.002), a lower Barthel Index (p < 0.001), a higher mRS score (p = 0.005), more frequently depressive symptoms (p = 0.042), and a larger lesion volume (p = 0.003) compared to patients with no/mild VCD. Cognitive scores are presented in Table 2. Supplementary Table 1 shows the correlation between lesion volume and cognitive performance of each domain within the VCD severity groups.

**Table 2:** Cognitive performance.

	Patients (n = 522)	^a^Cognitive impaired, n (%)
Z-score cognitive domain, mean (SD)		
Episodic memory	−0.7 (1.0)	112 (21.7)
Processing speed	−0.8 (1.1)	151 (29.1)
Visuoconstruction	−1.0 (0.9)	181 (36.4)
Executive functioning	−0.3 (0.8)	33 (6.3)
Visual neglect	0.3 (0.8)	33 (6.5)
Attention and working memory	−0.4 (0.7)	27 (5.4)
Language	−0.6 (0.9)	91 (18.3)
Aphasia, n (%)		
No aphasia	470 (90.6)	−
Mild to moderate aphasia	36 (6.9)	−
Severe aphasia	12 (2.3)	−
Mutism or global aphasia	1 (0.2)	−

SD: Standard Deviation; VCD: Vascular Cognitive Disorder. Missing data: episodic memory 6 (1.1 %); processing speed 3 (0.6 %); visuoconstruction 25 (4.8 %); visual neglect 11 (2.1 %), language 25 (4.8 %), attention and working memory 17 (3.3 %); aphasia 3 (0.6 %). ^a^ Percent cognitive impaired: the percentage of the patients with a Z-score of < -1.5.

### 4.1 Lesion coverage

**Fig. 2:**
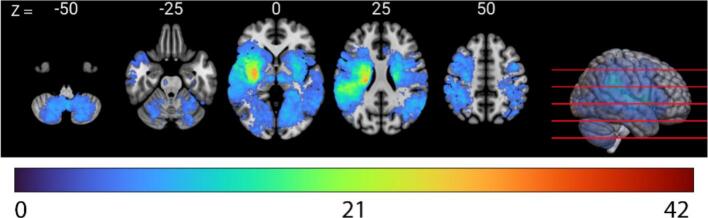
**Lesion overlap maps of all patients (n = 522).** Axial slices with a minimum overlap of 5 lesions. The images are presented in radiological orientation.

Lesion overlap maps are shown in Fig. 2. Patients most often had a lesion in the territory of the right middle cerebral artery (n = 187). The involvement of regions within the territories of the anterior and posterior cerebral artery, as well as portions of the brainstem and cerebellum, was too infrequent to allow for multivariate lesion-symptom mapping.

### 4.2 Multivariate lesion-symptom mapping

**Fig. 3:**
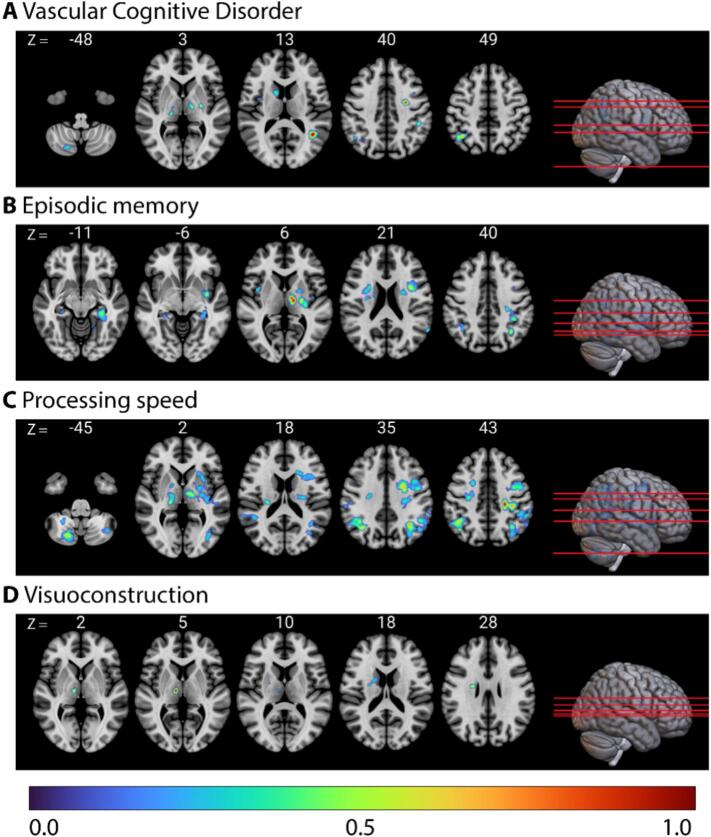

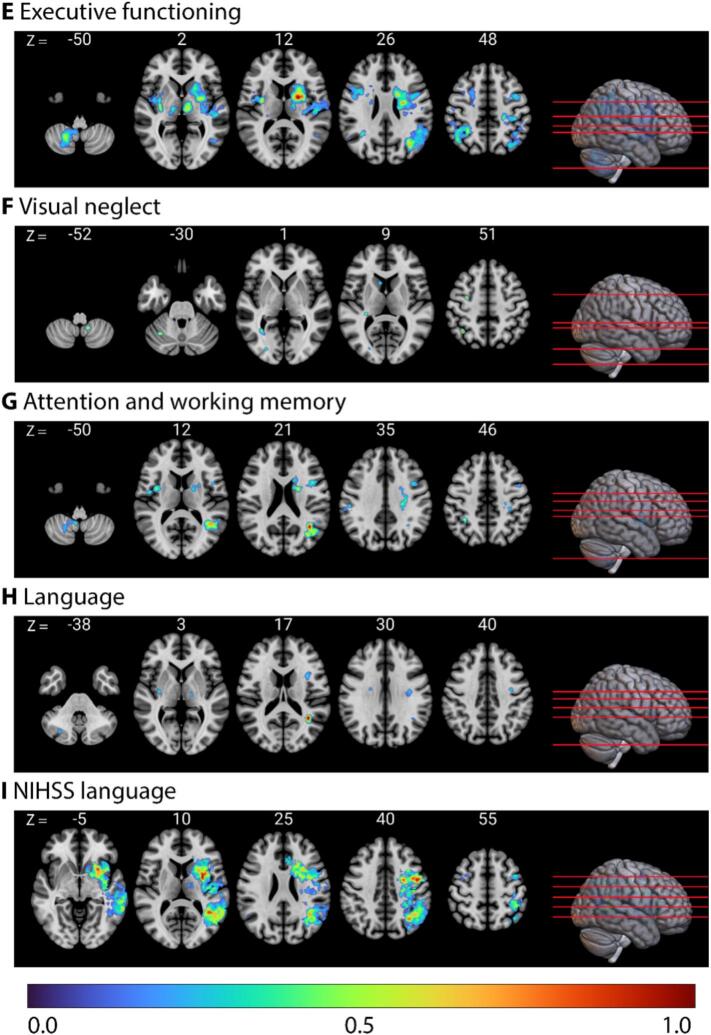
**Lesion-symptom mapping results for the presence of a major vascular cognitive disorder, each cognitive domain, and aphasia.** Axial slices in radiological orientation highlighting lesion locations associated with poorer cognitive performance. The color bar represents intensity values, ranging from 0 to 1, where 1 indicates the most robust relationship between the region and the cognitive score. (**A**) Vascular Cognitive Disorder. (**A**) Vascular Cognitive Disorder. (**B**) Episodic memory. (**C**) Processing speed. (**D**) Visuoconstruction. (**E**) Executive functioning. (**F**) Visual neglect. (**G**) Attention and working memory. (**H**) Language (**I**) NIHSS language subscale.

The LSM identified brain regions associated with poorer behavioral performance (Fig. 3). Supplementary Table 2 presents all identified peak regions associated with poorer performance, along with the peak ROI coordinates (X, Y, Z) for each cluster.

The presence of a major VCD was associated with regions in both hemispheres, with the strongest relationship observed in left angular gyrus. An additional cluster was located in the right cerebellum (r = 0.187, p < 0.001, optimal sparseness = 0.110).

The regions associated with poorer performance in episodic memory were mainly localized in bilateral frontal and hippocampal regions, left thalamus, and left insular regions (r = 0.168, p < 0.001, optimal sparseness = -0.271). For processing speed, regions associated with poorer performance were predominantly found in bilateral frontoparietal regions, left insular regions, and bilateral cerebellar regions (r = 0.222, p < 0.001, optimal sparseness = 0.485). Deep right frontal white matter and the right thalamus were associated with poorer performance in visuoconstruction (r = 0.092, p = 0.036, optimal sparseness = 0.019). For executive functioning, regions associated with poorer performance were mainly identified in bilateral frontoparietal and insular regions, with stronger relationships observed in the left hemisphere, and additionally, bilateral cerebellar regions (r = 0.277, p < 0.001, optimal sparseness = 0.637). Regions associated with visual neglect were right-lateralized, with the strongest relationships observed in small clusters in the right angular and precentral gyri. Additionally, there were regions located in the cerebellum (r = 0.167, p < 0.001, optimal sparseness = 0.027). Regions associated with poorer performance in attention and working memory were mainly located in bilateral parietal and insular regions, and left frontal regions, with an additional region the right cerebellum (r = 0.154, p < 0.001, optimal sparseness = -0.254).

For language, regions associated with poorer performance were mainly located in the left hemisphere, with the strongest relationship observed in a cluster in the left parietal white matter. An additional cluster was identified in the right cerebellum (r = 0.128, p = 0.003, optimal sparseness = 0.106). Finally, regions associated with aphasia, assessed with the NIHSS subscale, were predominantly left-lateralized (r = 0.626, p < 0.001, optimal sparseness = -0.456), and located in the fronto-temporo-parietal regions.

## 5 Sensitivity analyses

Sensitivity analyses correcting for lesion volume resulted in unexpected, reversed effects. After adjusting the cognitive outcomes for lesion volume, no significant clusters were observed for VCD, visual neglect, or language, while the results for other cognitive domains remained essentially similar. Adjusting the cognitive outcomes for the time interval between MRI and cognitive assessment and for depression prior to analysis did not essentially change our results. When restricting the analysis to early assessments (≤ 3 months), cluster focality was modestly reduced. No significant clusters remained for visuoconstruction or visual neglect.

## 6 Discussion

In this study, we show that lesion locations in different brain regions, often in different vascular distributions, are associated with poorer performance on various cognitive domains in young ischemic stroke patients. Additionally, we found that stroke lesions in the cerebellum, often overlooked in the context of stroke and cognition, were also associated with poorer cognitive performance in multiple cognitive domains. This widespread distribution showcases the complexity in the relationships between affected brain regions and cognitive symptoms, explaining the variability observed in young ischemic stroke patients with PSCI.

For most of the tested cognitive domains, including episodic memory, processing speed, executive functioning, language, attention and working memory, as well as for the presence of a major VCD, we identified regions in both hemispheres associated with poorer cognitive performance. While the overall distributions of the lesion locations associated with poorer cognitive performance appears widespread, the regions identified per cognitive domain correspond with established findings in the lesion literature. For example, involvement of the hippocampus, the prefrontal cortex, and the thalamus in episodic memory is well-documented (Eichenbaum, 2017). However, previous lesion-symptom mapping studies did not report bilateral brain regions associated with poorer cognitive performance across all these domains (Weaver et al., 2021, Bowren et al., 2022). In older participants with stable, focal stroke brain lesions in the chronic phase (three months or more after lesion onset), only left-hemispheric clusters were associated with worse performance in episodic memory and with language deficits (Bowren et al., 2022). One possibility for the difference with our study is that, performing imaging in the chronic phase, as that study did, may allow for more precise lesion segmentation, since diffusion restriction in the acute phase does not always indicate permanent tissue damage. This suggests that our findings could be less robust, as acute diffusion restriction does not reflect lasting structural damage. However, imaging from the chronic phase alone cannot fully account for the stronger left-lateralized associations with cognition. Another study, which included 12 cohorts of ischemic stroke patients and imaging in the acute phase, also found significant associations exclusively within the left hemisphere for verbal memory, language, and attention and executive functioning, using voxel-based lesion-symptom mapping (Weaver et al., 2021). It is possible that language deficits might have played a larger role in their findings, as only seven cohorts explicitly excluded patients with severe language impairment. This could potentially account for the observed lesion pattern typically associated with language. Consistent with our study, that study found regions in bilateral hemispheres predicting impairment in processing speed and overall PSCI, comparable with major VCD (Weaver et al., 2021). However, we found more smaller regions and less involvement of the left frontotemporal lobe in patients with major VCD. This difference, with our findings showing smaller regions compared to the voxel-based method, might be attributed to differences in analytical approaches, as multivariate methods are generally more accurate than mass-univariate approaches (Pustina et al., 2018). Additionally, the difference may also partly reflect variation in cut-off criteria for PSCI or major VCD. We applied a more conservative threshold for major VCD. These methodological differences may affect the size and severity of the major VCD or PSCI group and influence the lesion patterns identified. Another study involving 172 first-time stroke patients, of whom 80 % were older than 50 years, with imaging conducted one to two weeks post-stroke, demonstrated that lesion location explained more variance in motor and language deficits than in attention and memory impairments. In contrast to that study, we identified several small, distributed regions associated with poorer performance in attention and memory (Corbetta et al., 2015). This may suggest that, in patients with stroke at a young age, these associations are less confounded by other age-related cerebral comorbidities. Nevertheless, we concur with the authors that cognitive functions such as attention and memory likely depend on distributed patterns of activation.

In our study, the right angular and the right precentral gyrus were associated with neglect, consistent with the literature (Moore et al., 2023, Mort et al., 2003). This supports the relevance of these regions, but the identification of additional regions in other studies highlights the anatomical complexity and potentially diverse locations of brain regions involved in hemispatial neglect (Weaver et al., 2021, Moore et al., 2023, Biesbroek et al., 2014).

The NIHSS language subscale showed a strong brain-behavior relationship in our study. Specifically, left-sided fronto-temporo-parietal regions were associated with the presence of aphasia. This is in contrast with language deficits measured using cognitive tests, where we found a weak brain-behavior relationship and identified smaller clusters in various locations, including the cerebellum. The difference in the strength and the locations found in the different models might be due to the timing of the NIHSS, which was assessed in the acute phase along with the clinical MRI, whereas the cognitive tests were conducted mostly more than one-month post-stroke. The timing of cognitive assessment is relevant, as some recovery may occur during the first weeks after stroke, which may have reduced language deficits. In addition, the Short Token Test used for the language domain also requires attention and working memory, which may introduce variability unrelated to language impairment. Missing data in the language domain are unlikely to account for the observed difference between the strong lesion-behavior association for aphasia and the weaker association for language performance, as only 2 of the 13 patients with severe or global aphasia at discharge lacked a language domain score. Our testing at a median of 3 months aligns with other large cohorts (Weaver et al., 2021, Bowren et al., 2022). After this stage, we expect limited further cognitive improvement (Schellekens et al., 2023). Incorporating cognitive assessments in the acute phase, although challenging, could provide valuable insights into the association between PSCI and stroke location.

Interestingly, infratentorial lesions were also associated with the cognitive impairment in several cognitive domains. The cerebellum has often been a neglected region in earlier studies in relation to cognitive function. However, there is increasing evidence supporting its role in cognitive function (Schmahmann et al., 2019). Lesions of the posterior cerebellar lobe supposedly produce dysmetria of thought and emotion, also known as the cerebellar cognitive affective syndrome (Schmahmann et al., 2019). Our results align with a *meta*-analysis conducted in healthy adults, highlighting the role of cerebellar areas for language processing, especially in the right cerebellum (Turker et al., 2023). In addition, we found cerebellar regions associated with poorer performance for processing speed, executive functioning, visual neglect, language, attention and working memory, and the presence of a major VCD. This highlights the involvement of the cerebellum in cognitive processes. While this contrasts with findings with findings from a lesion-symptom mapping study (Weaver et al., 2021), which reported limited cerebellar involvement in post-stroke cognition, differences in cohort characteristics or analytic methods may underlie this discrepancy.

Our study has several strengths. First, this study includes a large prospective cohort of young adults who experienced a first-ever ischemic stroke, minimizing confounding factors like neurovascular and neurodegenerative changes, often seen in older adults. This provides a clearer view of stroke-induced cognitive changes. Second, we used extensive neuropsychological testing with minimal missing data. Finally, an important methodological strength is our use of multivariate LSM, which provides a more nuanced understanding of how specific brain regions contribute to cognitive outcomes.

However, some study limitations need to be addressed. First, cognitive data were lacking for patients who were unable to participate, for example due to severe aphasia. The underrepresentation of patients with a severe language impairment may have attenuated associations with language-related brain regions, potentially underestimating findings related to language deficits. Second, premorbid cognitive performance of our patients is unknown. However, all patients were under the age of 50, and we consider it unlikely that neurovascular changes, or neurodegenerative disorders substantially influenced our results. Third, we did not collect data on other interventions patients received, such as cognitive rehabilitation, which could have influenced cognitive recovery and impacted our results. Fourth, due to the multicenter design and extended recruitment period, multiple examiners administered the neuropsychological assessments, which may have introduced inter-observer variability, despite standardized training. To mitigate this potential observer bias, two trained researchers retrospectively reviewed all the cognitive assessments. Fifth, scanner differences across sites may have introduced variability in lesion delineation. Although all images were registered to standard space to minimize this effect, residual differences cannot be fully excluded. Sixth, we evaluated correction for lesion volume, a well-recognized confound in lesion-symptom mapping because of its strong correlation with lesion distribution and symptom severity (Pustina and Mirman, 2022). In our analyses using the internal LESYMAP option, lesion volume correction produced unexpected, reversed effects, with regions emerging as associated with better cognitive performance in anatomically unexpected locations, a known issue likely due to complex interrelations between lesion volume, location, and cognition. To further address this, we adjusted cognitive scores for lesion volume prior to lesion-symptom mapping. This strategy resulted in no significant clusters for VCD, visual neglect, or language, suggesting that these associations might be due to lesion volume. Given that other results remain unchanged, and consistent with recommendations that SCCAN can identify critical regions without lesion volume correction (Thye and Mirman, 2018), we opted to report uncorrected results. Seventh, the variance explained of most brain-behavior relationships, except for the NIHSS language subscale, was limited with r values ranging from 0.09 to 0.28. These effect sizes indicate that lesion location explains only a small proportion of variance in cognitive outcomes. These weak correlations may partly reflect the widespread distribution of the identified regions. Another possible explanation for the weak correlations could be the time gap between the initial MRI (performed median two days post-stroke) and the cognitive assessment (conducted at a median time point slightly less than three months post-stroke). This delay may result in a mismatch between the lesion location on MRI and cognitive functioning at three months, as early-stage diffusion restriction may not fully reflect its long-term impact, or recovery could alter the relationship over time. Importantly, lesion location is only one factor influencing post-stroke cognition. Other contributors include disruption of functional and structural brain networks (Evangelista et al., 2023), and individual differences in brain or cognitive reserve (Stern et al., 2023), which may buffer the impact of structural damage. These factors highlight the complexity of interpreting correlations between lesion location and cognitive outcome. Nonetheless, the use of acute stroke imaging is clinically relevant, as it provides early insights into which patients may be at risk for cognitive impairment.

In this study, we examined whether infarct locations in the acute phase were associated with PSCI after three months in young ischemic stroke patients. Future studies should investigate whether predictive modeling approaches, combining lesion location data with clinical and demographic variables and applying machine learning techniques, can capture these complex relationships and improve early risk stratification. The development of prediction models for PSCI, as earlier done for older patients (Weaver et al., 2021), might be useful to identify patients at risk for developing cognitive impairment at an early stage. This knowledge may support neurologists and other healthcare professionals to tailor individualized rehabilitation strategies that consider the variability in lesion location and their impact on different cognitive functions. However, such a prediction model would ideally be developed specifically for young ischemic stroke patients. Additionally, our findings primarily highlight focal regions associated with poorer cognitive performance. Future studies should explore whether these regions interact within broader neural networks. Such research could help clarify whether cognitive impairments result from focal lesions or network disruptions, and may offer a more integrated basis for prediction.

In conclusion, lesion locations associated with poorer cognitive performance in patients with stroke at a young age are widely distributed across various brain regions, with the specific cognitive deficits varying depending on the affected brain region. This widespread distribution showcases the complexity in the relationships between affected brain regions and cognitive symptoms, explaining the variability observed in young ischemic stroke patients with PSCI. Healthcare providers should be aware of this observation and integrate this knowledge into rehabilitation planning as well as when explaining the consequences of stroke to patients and their families. Future studies should focus on development of prediction models for PSCI in patients with stroke at a young age to improve personalized rehabilitation strategies.

## 7 Data Availability

Anonymized data not published within this article will be made available by request from any qualified investigator after permission of regulatory bodies and medical ethics committees.

## Declaration of Generative AI and AI-assisted technologies in the writing process

During the preparation of this manuscript, we used ChatGPT to refine the language. We carefully reviewed and edited all content afterwards and take full responsibility for the content of the published article.

## CRediT authorship contribution statement

**Mijntje M.I. Schellekens:** Writing – review & editing, Writing – original draft, Visualization, Methodology, Investigation, Formal analysis, Data curation, Conceptualization. **Hao Li:** Writing – review & editing, Methodology, Formal analysis. **Maartje Wijnands:** Writing – review & editing, Data curation. **Anastasia Papounidou:** Writing – review & editing, Data curation. **Esther M. Boot:** Writing – review & editing. **Jamie I. Verhoeven:** Writing – review & editing. **Merel S. Ekker:** Writing – review & editing. **Mayte E. van Alebeek:** Writing – review & editing. **Paul J.A.M. Brouwers:** Writing – review & editing. **Renate M. Arntz:** Writing – review & editing. **Gert W. van Dijk:** . **Rob A.R. Gons:** Writing – review & editing. **Inge W.M. van Uden:** . **Tom den Heijer:** Writing – review & editing. **Julia H. van Tuijl:** . **Karlijn F. de Laat:** Writing – review & editing. **Anouk G.W. van Norden:** . **Sarah E. Vermeer:** Writing – review & editing. **Marian S.G. van Zagten:** Writing – review & editing. **Robert J. van Oostenbrugge:** Writing – review & editing. **Marieke J.H. Wermer:** Writing – review & editing. **Paul J. Nederkoorn:** Writing – review & editing. **Frank G. van Rooij:** . **Ido R. van den Wijngaard:** Writing – review & editing. **Paul L.M. de Kort:** Writing – review & editing. **Vitória Piai:** Writing – review & editing. **Frank-Erik de Leeuw:** . **Roy P.C. Kessels:** Writing – review & editing. **Anil M. Tuladhar:** Writing – review & editing, Supervision, Project administration, Methodology, Funding acquisition, Conceptualization.

## Funding

This research received no specific grant from any funding agency in the public, commercial, or not-for-profit sectors.

## Declaration of Competing Interest

The authors declare the following financial interests/personal relationships which may be considered as potential competing interests: Marieke J. H. Wermer has received a VIDI grant (9171337) from ZonMw/NWO and the Clinical Established Investigator Dutch Heart Foundation grant (2016 T86), V. Piai is supported by the Netherlands Organization for Scientific Research (VI.Vidi.201.081), H. F. de Leeuw is a Clinical Established Investigator of the Dutch Heart Foundation (2014 T060), A. M. Tuladhar is a junior staff member of the Dutch Heart Foundation (grant number 2016 T044).

## Data Availability

Data will be made available on request.
