# Supplementary figures and images for: Lesion locations are associated with cognitive impairment after ischemic stroke in young adults
Source: Neuroimage Clin. 2025 Dec 17;49:103930. doi: 10.1016/j.nicl.2025.103930 (PMC12811597; doi:10.1016/j.nicl.2025.103930)

**Supplementary Figure**

*
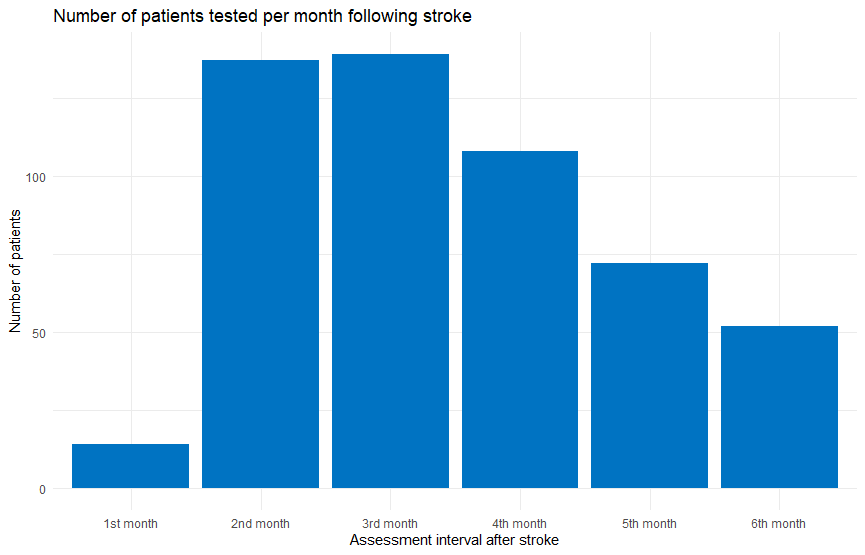
*

**Supplementary Figure 1 Distribution of cognitive assessment timing.**

Supplement: Supplementary Data 1 [file mmc1.docx]
