# Supplementary material for: Lesion locations are associated with cognitive impairment after ischemic stroke in young adults
Source: Neuroimage Clin. 2025 Dec 17;49:103930. doi: 10.1016/j.nicl.2025.103930 (PMC12811597; doi:10.1016/j.nicl.2025.103930)
Supplement: Supplementary Data 2 [file mmc2.docx]

**Supplementary** **Tables**

**Supplementary Table 1 Spearman’s rank correlations (ρ) between normalized lesion volume and cognitive performance within VCD severity groups.**

| **Cognitive domain** | **Spearman’s rho (ρ) no/mild VCD** | **p-value** | **FDR adjusted p-value** | **Spearman’s rho (ρ) major VCD** | **p-value** | **FDR adjusted p-value** |
| --- | --- | --- | --- | --- | --- | --- |
| Episodic memory | -0.03 | 0.599 | 0.599 | 0.004 | 0.962 | 0.962 |
| Processing speed | -0.04 | 0.426 | 0.597 | -0.19 | 0.013 | **0.046** |
| Visuoconstruction | -0.08 | 0.140 | 0.246 | -0.02 | 0.788 | 0.919 |
| Executive functioning | -0.18 | <0.001 | **0.004** | -0.22 | 0.004 | **0.031** |
| Visual neglect | -0.11 | 0.037 | 0.130 | -0.12 | 0.120 | 0.210 |
| Attention and working memory | 0.03 | 0.585 | 0.599 | -0.16 | 0.048 | 0.113 |
| Language | -0.09 | 0.106 | 0.246 | -0.04 | 0.604 | 0.845 |

VCD: Vascular Cognitive Disorder. P-values were adjusted for multiple comparisons using the Benjamini–Hochberg procedure (FDR). Significant adjusted p-values (FDR < 0.05) are shown in bold.

**Supplementary Table 2 Listing of the peak clusters for the presence of a major vascular cognitive disorder, each cognitive domain, and aphasia.** Anatomical location is the location to which each center of mass of the cluster belongs to. Intensity values ranging from 0 to 1, with 1 being the most robust positive relationship between the region and the inverse Z-score of each cognitive outcome, the presence of a vascular cognitive and the score of the NIHSS language subscale. Location of the peak ROI (X, Y, Z) for each cluster.

**VCD**

| **Cluster** | **Anatomical location** | **Number of voxels** | **Intensity** | **X** | **Y** | **Z** |
| --- | --- | --- | --- | --- | --- | --- |
|  | L Angular gyrus | 5410 | 0.996 | -43 | -54 | 13 |
|  | R Lateral occipital cortex, superior division | 3012 | 0.688 | 37 | -58 | 49 |
|  | L Cerebral white matter | 2487 | 0.884 | -29 | -7 | 40 |
|  | R Cerebral white matte | 961 | 0.614 | 31 | -2 | 18 |
|  | R Thalamus | 789 | 0.574 | 17 | -25 | 1 |
|  | R Cerebellum | 785 | 0.325 | 19 | -74 | -48 |
|  | R Caudate | 773 | 0.623 | 13 | 6 | 15 |
|  | L Thalamus | 764 | 0.501 | -14 | -14 | 4 |
|  | L Supramarginal gyrus, anterior division | 686 | 0.751 | -48 | -39 | 42 |
|  | L Middle frontal gyrus | 492 | 0.361 | -42 | 7 | 37 |
|  | L Putamen | 459 | 0.578 | -27 | -12 | 3 |

**Episodic memory**

| **Cluster** | **Anatomical location** | **Number of voxels** | **Intensity** | **X** | **Y** | **Z** |
| --- | --- | --- | --- | --- | --- | --- |
|  | L Putamen | 6434 | 0.599 | -25 | -15 | 6 |
|  | L Temporal fusiform cortex, posterior division | 5707 | 0.495 | -32 | -33 | -11 |
|  | L Cerebral white matter, L Inferior frontal gyrus | 4488 | 0.607 | -36 | 5 | 21 |
|  | R External capsule | 3296 | 0.523 | 28 | 1 | 18 |
|  | L Thalamus | 2726 | 0.970 | -14 | -14 | 8 |
|  | L Cerebral white matter, L Postcentral gyrus | 2515 | 0.569 | -36 | -31 | 45 |
|  | R Cerebral white matter, R Angular gyrus | 1447 | 0.290 | 35 | -55 | 32 |
|  | L Lateral occipital cortex, superior division | 1234 | 0.479 | -39 | -73 | 30 |
|  | L Angular gyrus | 1220 | 0.590 | -35 | -60 | 40 |
|  | L Cerebral white matter | 820 | 0.569 | -28 | -17 | 32 |
|  | L Cerebral white matter | 766 | 0.243 | -29 | -64 | 15 |
|  | R Hippocampus | 662 | 0.210 | 26 | -39 | -6 |
|  | R Angular gyrus | 336 | 0.321 | 51 | -58 | 32 |
|  | L Angular gyrus | 304 | 0.369 | -62 | -58 | 21 |
|  | L Lingual gyrus | 287 | 0.209 | -23 | -54 | -13 |
|  | L Angular gyrus | 287 | 0.254 | -50 | -57 | 15 |

**Processing speed**

| **Cluster** | **Anatomical location** | **Number of voxels** | **Intensity** | **X** | **Y** | **Z** |
| --- | --- | --- | --- | --- | --- | --- |
|  | L Superior corona radiata | 38002 | 0.979 | -25 | -27 | 44 |
|  | L Angular gyrus | 17859 | 0.718 | -35 | -57 | 35 |
|  | R Cerebral white matter, R precentral gyrus | 9371 | 0.522 | 24 | -25 | 16 |
|  | R Angular gyrus | 9292 | 0.709 | 42 | -53 | 41 |
|  | R Cerebellum | 3901 | 0.756 | 19 | -74 | -49 |
|  | L Lateral occipital cortex, inferior division | 2408 | 0.359 | -41 | -73 | 1 |
|  | R Thalamus | 1868 | 0.518 | 17 | -24 | 2 |
|  | R Angular gyrus | 1506 | 0.384 | 46 | -49 | 21 |
|  | R Middle temporal gyrus, temporooccipital part | 1152 | 0.364 | 60 | -58 | 9 |
|  | L Cerebellum | 1030 | 0.319 | -42 | -66 | -43 |
|  | R Cerebellum | 820 | 0.306 | 25 | -53 | -46 |
|  | R Superior temporal gyrus, posterior division | 718 | 0.177 | 50 | -31 | -2 |
|  | R Supramarginal gyrus, anterior division | 532 | 0.244 | 59 | -31 | 36 |
|  | L Hippocampus | 463 | 0.241 | -33 | -29 | -14 |
|  | Brain-stem | 421 | 0.284 | 5 | -23 | -31 |
|  | R Pallidum | 302 | 0.234 | 15 | 0 | 2 |
|  | R Lateral occipital cortex, inferior division | 286 | 0.195 | 49 | -74 | 11 |

**Visuoconstruction**

| **Cluster** | **Anatomical location** | **Number of voxels** | **Intensity** | **X** | **Y** | **Z** |
| --- | --- | --- | --- | --- | --- | --- |
|  | R Superior fronto-occipital fasciculus | 2072 | 0.630 | 23 | -9 | 28 |
|  | R Thalamus | 813 | 0.943 | 10 | -15 | 5 |

**Executive functioning**

| **Cluster** | **Anatomical location** | **Number of voxels** | **Intensity** | **X** | **Y** | **Z** |
| --- | --- | --- | --- | --- | --- | --- |
|  | L Putamen | 54028 | 0.982 | -22 | 0 | 11 |
|  | L Angular gyrus | 29139 | 0.700 | -37 | -71 | 26 |
|  | R Cerebral white matter, R Central opercular cortex | 24245 | 0.674 | 32 | -5 | 12 |
|  | R Superior parietal lobe | 16925 | 0.576 | 29 | -49 | 49 |
|  | R Cerebellum | 13480 | 0.503 | 20 | -59 | -50 |
|  | L Cerebral white matter, L Postcentral gyrus | 4029 | 0.619 | -25 | -29 | 44 |
|  | R Thalamus | 1759 | 0.410 | 13 | -17 | 2 |
|  | L Middle temporal gyrus, posterior division | 1724 | 0.277 | -63 | -24 | -10 |
|  | Brain-stem | 913 | 0.722 | 4 | -23 | -30 |
|  | R Retrolenticular part of internal capsule | 792 | 0.356 | 27 | -27 | 19 |
|  | R Postcentral gyrus | 670 | 0.194 | 51 | -18 | 48 |
|  | R Cerebral white matter, R Hippocampus | 564 | 0.168 | 28 | -27 | -16 |
|  | L Lateral occipital cortex, inferior division | 456 | 0.232 | -49 | -64 | 2 |
|  | L Cerebellum | 340 | 0.187 | -26 | -57 | -23 |
|  | R Lingual gyrus | 340 | 0.167 | 19 | -43 | -7 |

**Visual neglect**

| **Cluster** | **Anatomical location** | **Number of voxels** | **Intensity** | **X** | **Y** | **Z** |
| --- | --- | --- | --- | --- | --- | --- |
|  | R Angular gyrus | 887 | 0.970 | 37 | -57 | 49 |
|  | R Cerebellum | 510 | 0.590 | 31 | -62 | -30 |
|  | R Posterior thalamic radiation | 486 | 0.527 | 32 | -57 | 0 |
|  | L Cerebellum | 470 | 0.679 | -13 | -52 | -52 |
|  | R Precentral gyrus | 416 | 0.972 | 31 | -8 | 52 |
|  | R Caudate | 382 | 0.311 | 8 | 13 | 9 |
|  | R cerebral white matter, R Lateral occipital cortex, superior division | 367 | 0.370 | 25 | -84 | 1 |
|  | R Cerebral white matter | 343 | 0.487 | 29 | -32 | 8 |

**Attention and working memory**

| **Cluster** | **Anatomical location** | **Number of voxels** | **Intensity** | **X** | **Y** | **Z** |
| --- | --- | --- | --- | --- | --- | --- |
|  | L Superior corona radiata | 8539 | 0.454 | -22 | -2 | 22 |
|  | L Angular gyrus | 8351 | 0.724 | -39 | -57 | 22 |
|  | L Inferior frontal gyrus | 2887 | 0.326 | -43 | 8 | 20 |
|  | R Insular cortex | 2648 | 0.329 | 37 | -1 | 12 |
|  | R Cerebellum | 2479 | 0.278 | 5 | -53 | -50 |
|  | R Superior parietal lobe | 898 | 0.400 | 33 | -47 | 46 |
|  | L Cerebral white matter, L Insular cortex | 688 | 0.263 | -29 | 20 | 8 |
|  | R Cerebral white matter, R Supramarginal gyrus, anterior division | 665 | 0.240 | 53 | -27 | 35 |

**Language**

| **Cluster** | **Anatomical location** | **Number of voxels** | **Intensity** | **X** | **Y** | **Z** |
| --- | --- | --- | --- | --- | --- | --- |
|  | L Cerebral white matter, L Supramarginal gyrus, posterior division | 1882 | 0.993 | -38 | -52 | 17 |
|  | L Frontal operculum cortex | 1586 | 0.422 | -36 | 8 | 15 |
|  | L Superior longitudinal fasciculus | 828 | 0.411 | -31 | -16 | 33 |
|  | L Thalamus | 819 | 0.594 | -15 | -15 | 8 |
|  | R Cerebral white matter, R Caudate | 602 | 0.302 | 21 | -10 | 28 |
|  | R Putamen | 371 | 0.333 | 32 | -13 | 3 |
|  | R Cerebellum | 366 | 0.294 | 28 | -72 | -38 |
|  | L Cerebral white matter, L Precentral gyrus | 327 | 0.312 | -35 | -9 | 40 |

**NIHSS language subscale at discharge**

| **Cluster** | **Anatomical location** | **Number of voxels** | **Intensity** | **X** | **Y** | **Z** |
| --- | --- | --- | --- | --- | --- | --- |
|  | L Parietal operculum cortex | 156580 | 0.980 | -47 | -3 | 39 |
|  | R Lateral occipital cortex | 1159 | 0.307 | 52 | -58 | 16 |
|  | R Middle frontal gyrus | 946 | 0.137 | 30 | 2 | 55 |
