# Supplementary material for: Lesion locations are associated with cognitive impairment after ischemic stroke in young adults
Source: Neuroimage Clin. 2025 Dec 17;49:103930. doi: 10.1016/j.nicl.2025.103930 (PMC12811597; doi:10.1016/j.nicl.2025.103930)
Supplement: Supplementary Data 3 [file mmc3.docx]

**Supplementary Methods**

**Participating centers of the ODYSSEY study**

1. Radboud University Medical Centre, Nijmegen, the Netherlands
2. Medisch Spectrum Twente, Enschede, the Netherlands
3. Canisius-Wilhelmina Hospital, Nijmegen, the Netherlands
4. Catharina Hospital, Eindhoven, the Netherlands
5. Franciscus Gasthuis & Vlietland, Rotterdam, the Netherlands
6. Elisabeth-TweeSteden Hospital, Tilburg, the Netherlands
7. Haga Hospital, Den Haag, Netherlands
8. Amphia Hospital, Breda, the Netherlands
9. Rijnstate Hospital, Arnhem, the Netherlands
10. Jeroen Bosch Hospital, 's-Hertogenbosch, the Netherlands
11. Maastricht University Medical Centre, Maastricht, the Netherlands
12. Leiden University Medical Centre, Leiden, the Netherlands
13. Amsterdam University Medical Centre, location AMC, Amsterdam, the Netherlands
14. Albert Schweitzer Hospital, Dordrecht, The Netherlands
15. Zuyderland Hospital, Sittard-Geleen, the Netherlands
16. Medical Centre Leeuwarden, Leeuwarden, the Netherlands
17. Haaglanden Medical Centre, Den Haag, The Netherlands

**Cognitive assessment**We assessed seven cognitive domains using multiple tests: *(i) Episodic memory* (3-trial version of the Rey Auditory Verbal Learning Test), (ii) *Processing speed* (the written version of the Symbol-Digit Modalities Test, the abbreviated Stroop Color Word Test, parts I and II), (iii) *Visuoconstruction* (Rey-Osterrieth Complex Figure (ROCF)-copy trial), (iv) *Executive functioning* (Fluency test, Stroop interference score, Brixton Spatial Anticipation Test), (v) *Visual neglect* (Star Cancellation of the Behavioral Inattention Test), (vi) *Attention and working memory* (Digit Span subtest from the Wechsler adult Intelligence Scale – Fourth Edition), and (vii) *Language* (Short Token Test). Normative data from the Advanced Neuropsychological Diagnostics Infrastructure (ANDI), which includes data of 26,000 healthy individuals across all age groups were employed for most tests. This allowed fine-grained adjustment based on age, sex and education level. For the written version of the Symbol-Digit Modalities Test (Smit A, 2010), we used the normative data from the test’s manual. We used normative data of healthy controls from another stroke study for the Star Cancellation test (Nys GM et al., 2006).

**The MRI scanners and their respective scanning parameters per scanner**

| 1 | 1.5T Philips. Imaging protocols: (I) DWI (TR/TE 3157/80 ms; voxel size 1.3 × 1.3 × 6 mm) |
| --- | --- |
| 2 | 3T GE. Imaging protocols: (I) T1 (TR/TE 560/10 ms; voxel size 0.5 × 0.5 × 4.5 mm); (II) FLAIR (TR/TE 12000/99 ms; voxel size 0.5 × 0.5 × 4.5 mm); (III) DWI (TR/TE 4600/72 ms; voxel size 0.9 × 0.9 × 4.5 mm) |
| 3 | 3T GE. Imaging protocols: (I) DWI (TR/TE 4875/85 ms; voxel size 0.9 × 0.9 × 4.4 mm) |
| 4 | 1.5T GE. Imaging protocols: (I) FLAIR (TR/TE 9368/120 ms; voxel size 0.5 × 0.5 × 4.5 mm); (II) DWI (TR/TE 4800/68 ms; voxel size 0.9 × 0.9 × 4.5 mm) |
| 5 | 3T GE. Imaging protocols: (I) FLAIR (TR/TE 12000/99 ms; voxel size 0.5 × 0.5 × 4.5 mm); (II) DWI (TR/TE 4600/72 ms; voxel size 0.9 × 0.9 × 4.5 mm) |
| 6 | 1T Philips. Imaging protocols: (I) T1 (TR/TE 567/11 ms; voxel size 0.9 × 0.9 × 5.5 mm); (II) FLAIR (TR/TE 8000/100 ms; voxel size 0.8 × 0.8 × 5.5 mm); (III) DWI (TR/TE 4455/76 ms; voxel size 0.9 × 0.9 × 5.5 mm) |
| 7 | 1.5T Philips. Imaging protocols: (I) FLAIR (TR/TE 8000/120 ms; voxel size 0.4 × 0.4 × 5.5 mm); (II) DWI (TR/TE 4348/89 ms; voxel size 2 × 2 × 6 mm) |
| 8 | 1.5T Philips. Imaging protocols: (I) FLAIR (TR/TE 10000/140 ms; voxel size 0.8 × 0.8 × 5.5 mm); (II) DWI (TR/TE 5168/72 ms; voxel size 0.9 × 0.9 × 3.3 mm) |
| 9 | 1.5T Philips. Imaging protocols: (I) T1 (TR/TE 624/15 ms; voxel size 0.9 × 0.9 × 5.5 mm); (II) FLAIR (TR/TE 8000/140 ms; voxel size 0.9 × 0.9 × 6 mm); (III) DWI (TR/TE 3528/89 ms; voxel size 0.9 × 0.9 × 5.5 mm) |
| 10 | 1.5T Philips. Imaging protocols: (I) T1 (TR/TE 625/15 ms; voxel size 0.9 × 0.9 × 6.1 mm); (II) FLAIR (TR/TE 11000/140 ms; voxel size 0.9 × 0.9 × 6 mm); (III) DWI (TR/TE 3069/75 ms; voxel size 0.9 × 0.9 × 6.1 mm) |
| 11 | 1.5T Philips. Imaging protocols: (I) T1 (TR/TE 568/15 ms; voxel size 0.9 × 0.9 × 6 mm); (II) FLAIR (TR/TE 6000/120 ms; voxel size 0.9 × 0.9 × 6 mm); (III) DWI (TR/TE 3776/91 ms; voxel size 0.9 × 0.9 × 6 mm) |
| 12 | 1.5T Philips. Imaging protocols: (I) T1 (TR/TE 596/15 ms; voxel size 0.9 × 0.9 × 6 mm); (II) FLAIR (TR/TE 6000/120 ms; voxel size 0.9 × 0.9 × 6 mm); (III) DWI (TR/TE 3045/74 ms; voxel size 1.2 × 1.2 × 6 mm) |
| 13 | 3T Philips. Imaging protocols: (I) FLAIR (TR/TE 11000/125 ms; voxel size 0.4 × 0.4 × 5.5 mm); (II) DWI (TR/TE 2675/72 ms; voxel size 0.9 × 0.9 × 5.5 mm) |
| 14 | 3T Philips. Imaging protocols: (I) FLAIR (TR/TE 11000/125 ms; voxel size 0.4 × 0.4 × 5.5 mm); (II) DWI (TR/TE 10000/68 ms; voxel size 0.9 × 0.9 × 5.5 mm) |
| 15 | 3T Philips. Imaging protocols: (I) FLAIR (TR/TE 11000/120 ms; voxel size 0.6 × 0.6 × 5 mm); (II) DWI (TR/TE 3637/84 ms; voxel size 1.2 × 1.2 × 5 mm) |
| 16 | 1.5T Philips. Imaging protocols: (I) T1 (TR/TE 623/15 ms; voxel size 0.9 × 0.9 × 6 mm); (II) FLAIR (TR/TE 6000/120 ms; voxel size 0.9 × 0.9 × 6 mm); (III) DWI (TR/TE 3621/90 ms; voxel size 0.9 × 0.9 × 6 mm) |
| 17 | 1T Philips. Imaging protocols: (I) T1 (TR/TE 674/16 ms; voxel size 1 × 1 × 5.5 mm); (II) FLAIR (TR/TE 11000/140 ms; voxel size 0.7 × 0.7 × 5.5 mm) |
| 18 | 1.5T Philips. Imaging protocols: (I) T1 (TR/TE 596/15 ms; voxel size 0.9 × 0.9 × 6 mm); (II) FLAIR (TR/TE 6000/120 ms; voxel size 0.9 × 0.9 × 6 mm); (III) DWI (TR/TE 4271/114 ms; voxel size 1.2 × 1.2 × 6 mm) |
| 19 | 1.5T Philips. Imaging protocols: (I) T1 (TR/TE 568/15 ms; voxel size 0.9 × 0.9 × 6 mm); (II) FLAIR (TR/TE 6000/120 ms; voxel size 0.9 × 0.9 × 6 mm); (III) DWI (TR/TE 3101/88 ms; voxel size 0.9 × 0.9 × 6 mm) |
| 20 | 1.5T Philips. Imaging protocols: (I) T1 (TR/TE 624/15 ms; voxel size 0.9 × 0.9 × 5.5 mm); (II) FLAIR (TR/TE 8000/140 ms; voxel size 0.9 × 0.9 × 6 mm); (III) DWI (TR/TE 3309/89 ms; voxel size 0.9 × 0.9 × 5.5 mm) |
| 21 | 1.5T Philips. Imaging protocols: (I) T1 (TR/TE 621/15 ms; voxel size 0.5 × 0.5 × 5.5 mm); (II) FLAIR (TR/TE 6000/120 ms; voxel size 0.9 × 0.9 × 5.5 mm); (III) DWI (TR/TE 3735/72 ms; voxel size 1.2 × 1.2 × 6 mm) |
| 22 | 1.5T Philips. Imaging protocols: (I) FLAIR (TR/TE 6000/120 ms; voxel size 0.9 × 0.9 × 6 mm); (II) DWI (TR/TE 3238/81 ms; voxel size 1.4 × 1.4 × 6 mm) |
| 23 | 3T Philips. Imaging protocols: (I) T1 (TR/TE 500/10 ms; voxel size 1 × 0.9 × 0.9 mm); (II) FLAIR (TR/TE 11000/125 ms; voxel size 0.4 × 0.4 × 6 mm); (III) DWI (TR/TE 3927/94 ms; voxel size 0.9 × 0.9 × 6 mm) |
| 24 | 1.5T Siemens. Imaging protocols: (I) T1 (TR/TE 532/10 ms; voxel size 0.6 × 0.6 × 5.5 mm); (II) FLAIR (TR/TE 9000/108 ms; voxel size 0.7 × 0.7 × 5.5 mm); (III) DWI (TR/TE 6175/89 ms; voxel size 1.2 × 1.2 × 5.5 mm) |
| 25 | 1.5T Siemens. Imaging protocols: (I) T1 (TR/TE 532/10 ms; voxel size 0.6 × 0.6 × 5.5 mm); (II) FLAIR (TR/TE 9000/108 ms; voxel size 0.7 × 0.7 × 5.5 mm); (III) DWI (TR/TE 6175/89 ms; voxel size 1.2 × 1.2 × 5.5 mm) |
| 26 | 1.5T Siemens. Imaging protocols: (I) T1 (TR/TE 420/9 ms; voxel size 0.3 × 0.3 × 6.5 mm); (II) FLAIR (TR/TE 9000/98 ms; voxel size 0.4 × 0.4 × 6.5 mm); (III) DWI (TR/TE 3012/114 ms; voxel size 1.2 × 1.2 × 6.5 mm) |
| 27 | 1.5T Siemens. Imaging protocols: (I) FLAIR (TR/TE 9000/89 ms; voxel size 0.5 × 0.5 × 6.5 mm); (II) DWI (TR/TE 4970/114 ms; voxel size 2 × 2 × 6 mm) |
| 28 | 1.5T Siemens. Imaging protocols: (I) T1 (TR/TE 550/8 ms; voxel size 0.4 × 0.4 × 4.8 mm); (II) FLAIR (TR/TE 9000/87 ms; voxel size 0.4 × 0.4 × 5.8 mm); (III) DWI (TR/TE 2334/94 ms; voxel size 1.2 × 1.2 × 6.5 mm) |
| 29 | 1.5T Siemens. Imaging protocols: (I) T1 (TR/TE 450/9 ms; voxel size 0.5 × 0.5 × 6.5 mm); (II) FLAIR (TR/TE 9000/92 ms; voxel size 0.4 × 0.4 × 6.5 mm); (III) DWI (TR/TE 3389/102 ms; voxel size 1.2 × 1.2 × 6.5 mm) |
| 30 | 1.5T Siemens. Imaging protocols: (I) T1 (TR/TE 550/8 ms; voxel size 0.4 × 0.4 × 6.5 mm); (II) FLAIR (TR/TE 9000/89 ms; voxel size 0.4 × 0.4 × 6.5 mm); (III) DWI (TR/TE 2410/89 ms; voxel size 1.2 × 1.2 × 6.5 mm) |
| 31 | 1.5T Siemens. Imaging protocols: (I) T1 (TR/TE 470/8 ms; voxel size 0.4 × 0.4 × 6.5 mm); (II) DWI (TR/TE 2786/89 ms; voxel size 1.2 × 1.2 × 6.5 mm) |
| 32 | 1.5T Siemens. Imaging protocols: (I) T1 (TR/TE 1900/3 ms; voxel size 1 × 0.5 × 0.5 mm); (II) FLAIR (TR/TE 9000/109 ms; voxel size 0.4 × 0.4 × 6.5 mm); (III) DWI (TR/TE 3500/87 ms; voxel size 1.4 × 1.4 × 6.5 mm) |
| 33 | 1.5T Siemens. Imaging protocols: (I) T1 (TR/TE 470/9 ms; voxel size 0.4 × 0.4 × 6.5 mm); (II) FLAIR (TR/TE 9000/92 ms; voxel size 0.4 × 0.4 × 6.5 mm); (III) DWI (TR/TE 3088/102 ms; voxel size 1.2 × 1.2 × 6.5 mm) |
| 34 | 1.5T Siemens. Imaging protocols: (I) T1 (TR/TE 474/12 ms; voxel size 0.4 × 0.4 × 6.5 mm); (II) FLAIR (TR/TE 7000/89 ms; voxel size 0.4 × 0.4 × 6.5 mm); (III) DWI (TR/TE 3539/113 ms; voxel size 1.2 × 1.2 × 6.5 mm) |
| 35 | 1.5T Siemens. Imaging protocols: (I) FLAIR (TR/TE 9000/107 ms; voxel size 0.9 × 0.9 × 6 mm); (II) DWI (TR/TE 7229/111 ms; voxel size 1.2 × 1.2 × 6 mm) |
| 36 | 3T Siemens. Imaging protocols: (I) T1 (TR/TE 500/9 ms; voxel size 0.7 × 0.7 × 5.5 mm); (II) FLAIR (TR/TE 9000/86 ms; voxel size 0.6 × 0.6 × 5.5 mm); (III) DWI (TR/TE 3162/91 ms; voxel size 1.2 × 1.2 × 5.5 mm) |
| 37 | 1.5T Siemens. Imaging protocols: (I) T1 (TR/TE 510/9 ms; voxel size 0.5 × 0.5 × 6 mm); (II) FLAIR (TR/TE 9000/106 ms; voxel size 0.5 × 0.5 × 6 mm); (III) DWI (TR/TE 6928/105 ms; voxel size 1.2 × 1.2 × 6 mm) |
| 38 | 3T Philips. Imaging protocols: (I) T1 (TR/TE 10/5 ms; voxel size 3 × 0.7 × 0.7 mm); (II) FLAIR (TR/TE 11000/125 ms; voxel size 0.4 × 0.4 × 5 mm); (III) DWI (TR/TE 2736/59 ms; voxel size 1.2 × 1.2 × 5 mm) |
| 39 | 3T Siemens. Imaging protocols: (I) DWI (TR/TE 9940/100 ms; voxel size 1.1 × 1.1 × 5.2 mm) |
| 40 | 1.5T Philips. Imaging protocols: (I) FLAIR (TR/TE 6000/120 ms; voxel size 0.7 × 0.7 × 6 mm); (II) DWI (TR/TE 3747/106 ms; voxel size 1.3 × 1.3 × 6 mm) |
| 41 | 1.5T Philips. Imaging protocols: (I) T1 (TR/TE 500/12 ms; voxel size 0.6 × 0.6 × 6.5 mm); (II) FLAIR (TR/TE 11000/140 ms; voxel size 0.5 × 0.5 × 6 mm); (III) DWI (TR/TE 3406/99 ms; voxel size 1 × 1 × 6 mm) |
| 42 | 1.5T Philips. Imaging protocols: (I) T1 (TR/TE 504/12 ms; voxel size 0.7 × 0.7 × 6 mm); (II) FLAIR (TR/TE 6000/120 ms; voxel size 0.7 × 0.7 × 6 mm); (III) DWI (TR/TE 3570/111 ms; voxel size 1.3 × 1.3 × 6 mm) |
| 43 | 1.5T Siemens. Imaging protocols: (I) T1 (TR/TE 400/10 ms; voxel size 0.7 × 0.7 × 6.5 mm); (II) FLAIR (TR/TE 8000/86 ms; voxel size 0.7 × 0.7 × 5.2 mm); (III) DWI (TR/TE 105215/72 ms; voxel size 1.4 × 1.4 × 5.2 mm) |
| 44 | 1.5T Philips. Imaging protocols: (I) FLAIR (TR/TE 6000/120 ms; voxel size 0.7 × 0.7 × 6 mm); (II) DWI (TR/TE 3773/107 ms; voxel size 1.3 × 1.3 × 6 mm) |
| 45 | 1.5T Philips. Imaging protocols: (I) T1 (TR/TE 178/2 ms; voxel size 0.9 × 0.9 × 5.5 mm); (II) FLAIR (TR/TE 8000/120 ms; voxel size 0.5 × 0.5 × 5.5 mm); (III) DWI (TR/TE 4130/104 ms; voxel size 1.3 × 1.3 × 6 mm) |
| 46 | 1.5T Philips. Imaging protocols: (I) T1 (TR/TE 581/15 ms; voxel size 0.9 × 0.9 × 6 mm); (II) FLAIR (TR/TE 8500/120 ms; voxel size 0.7 × 0.7 × 6 mm); (III) DWI (TR/TE 3006/88 ms; voxel size 1 × 1 × 6 mm) |
| 47 | 1.5T Siemens. Imaging protocols: (I) T1 (TR/TE 1900/3 ms; voxel size 1 × 1 × 1 mm); (II) FLAIR (TR/TE 9000/89 ms; voxel size 0.7 × 0.7 × 4.4 mm); (III) DWI (TR/TE 6800/89 ms; voxel size 1.2 × 1.2 × 5.5 mm) |
| 48 | 1.5T Siemens. Imaging protocols: (I) T1 (TR/TE 550/9 ms; voxel size 0.7 × 0.7 × 6.5 mm); (II) FLAIR (TR/TE 9000/84 ms; voxel size 0.7 × 0.7 × 6.5 mm); (III) DWI (TR/TE 6300/89 ms; voxel size 1.2 × 1.2 × 6.5 mm) |
| 49 | 1.5T Siemens. Imaging protocols: (I) T1 (TR/TE 413/9 ms; voxel size 0.7 × 0.7 × 5.5 mm); (II) FLAIR (TR/TE 9000/86 ms; voxel size 0.4 × 0.4 × 5.5 mm); (III) DWI (TR/TE 7300/89 ms; voxel size 0.6 × 0.6 × 5.5 mm) |
| 50 | 1.5T Siemens. Imaging protocols: (I) FLAIR (TR/TE 9000/86 ms; voxel size 0.7 × 0.7 × 6.5 mm); (II) DWI (TR/TE 7100/89 ms; voxel size 0.6 × 0.6 × 6.5 mm) |
| 51 | 1.5T Siemens. Imaging protocols: (I) T1 (TR/TE 450/9 ms; voxel size 0.7 × 0.7 × 6.5 mm); (II) FLAIR (TR/TE 9750/82 ms; voxel size 0.7 × 0.7 × 6.5 mm); (III) DWI (TR/TE 4125/115 ms; voxel size 1.2 × 1.2 × 6.5 mm) |
| 52 | 3T Philips. Imaging protocols: (I) T1 (TR/TE 678/24 ms; voxel size 0.4 × 0.4 × 4 mm); (II) FLAIR (TR/TE 11000/125 ms; voxel size 0.4 × 0.4 × 5.5 mm); (III) DWI (TR/TE 2779/73 ms; voxel size 1.1 × 1.1 × 5.5 mm) |
| 53 | 3T Philips. Imaging protocols: (I) T1 (TR/TE 710/9 ms; voxel size 0.6 × 0.6 × 3.3 mm); (II) DWI (TR/TE 6440/83 ms; voxel size 0.9 × 0.9 × 3.3 mm) |
| 54 | 3T Philips. Imaging protocols: (I) T1 (TR/TE 600/10 ms; voxel size 0.5 × 0.5 × 5.5 mm); (II) FLAIR (TR/TE 11000/125 ms; voxel size 0.4 × 0.4 × 5.5 mm); (III) DWI (TR/TE 3233/98 ms; voxel size 0.9 × 0.9 × 5.5 mm) |
| 55 | 3T Philips. Imaging protocols: (I) T1 (TR/TE 500/10 ms; voxel size 0.5 × 0.5 × 5.5 mm); (II) FLAIR (TR/TE 4800/120 ms; voxel size 0.6 × 1 × 1 mm); (III) DWI (TR/TE 3188/85 ms; voxel size 0.9 × 0.9 × 3.3 mm) |
| 56 | 3T Philips. Imaging protocols: (I) T1 (TR/TE 10/6 ms; voxel size 0.6 × 0.6 × 2 mm); (II) FLAIR (TR/TE 4800/309 ms; voxel size 1 × 1 × 2 mm); (III) DWI (TR/TE 3325/91 ms; voxel size 1 × 1 × 6 mm) |
| 57 | 3T Siemens. Imaging protocols: (I) T1 (TR/TE 500/9 ms; voxel size 0.7 × 0.7 × 5.5 mm); (II) FLAIR (TR/TE 9000/87 ms; voxel size 0.6 × 0.6 × 5.5 mm); (III) DWI (TR/TE 5250/93 ms; voxel size 1.2 × 1.2 × 5.5 mm) |
| 58 | 3T Siemens. Imaging protocols: (I) T1 (TR/TE 1900/3 ms; voxel size 0.4 × 0.4 × 1 mm); (II) FLAIR (TR/TE 9000/81 ms; voxel size 0.7 × 0.7 × 3.3 mm); (III) DWI (TR/TE 6800/98 ms; voxel size 1.2 × 1.2 × 4.9 mm) |
| 59 | 3T Siemens. Imaging protocols: (I) T1 (TR/TE 440/2 ms; voxel size 0.7 × 0.7 × 5.2 mm); (II) FLAIR (TR/TE 440/2 ms; voxel size 0.7 × 0.7 × 5.2 mm); (III) DWI (TR/TE 132312/73 ms; voxel size 1 × 1 × 5.2 mm) |
| 60 | 1.5T Philips. Imaging protocols: (I) T1 (TR/TE 660/15 ms; voxel size 0.8 × 0.8 × 6 mm); (II) DWI (TR/TE 3190/85 ms; voxel size 1.2 × 1.2 × 6 mm) |
| 61 | 1.5T Siemens. Imaging protocols: (I) T1 (TR/TE 15/5 ms; voxel size 1 × 1 × 1 mm); (II) DWI (TR/TE 3700/105 ms; voxel size 1.4 × 1.4 × 6.5 mm) |
| 62 | 1.5T Siemens. Imaging protocols: (I) T1 (TR/TE 2020/5 ms; voxel size 0.5 × 0.5 × 1 mm); (II) FLAIR (TR/TE 9000/91 ms; voxel size 0.4 × 0.4 × 6.5 mm); (III) DWI (TR/TE 8400/129 ms; voxel size 1.2 × 1.2 × 6.5 mm) |
| 63 | 3T Siemens. Imaging protocols: (I) T1 (TR/TE 2300/2 ms; voxel size 0.9 × 0.9 × 0.9 mm); (II) FLAIR (TR/TE 5000/394 ms; voxel size 1 × 0.5 × 0.5 mm); (III) DWI (TR/TE 6900/67 ms; voxel size 2 × 2 × 2 mm) |
| 64 | 1.5T Siemens. Imaging protocols: (I) T1 (TR/TE 758/14 ms; voxel size 0.7 × 0.7 × 9.9 mm) |
| 65 | 1.5T Philips. Imaging protocols: (I) T1 (TR/TE 10/5 ms; voxel size 0.8 × 0.8 × 1 mm); (II) FLAIR (TR/TE 11000/130 ms; voxel size 0.4 × 0.4 × 5.5 mm); (III) DWI (TR/TE 3346/80 ms; voxel size 0.9 × 0.9 × 5.5 mm) |
| 66 | 1.5T Philips. Imaging protocols: (I) T1 (TR/TE 660/15 ms; voxel size 0.9 × 0.9 × 5.5 mm); (II) FLAIR (TR/TE 4800/299 ms; voxel size 0.6 × 1 × 1 mm); (III) DWI (TR/TE 4864/89 ms; voxel size 0.9 × 0.9 × 3.3 mm) |
| 67 | 1.5T Philips. Imaging protocols: (I) T1 (TR/TE 607/15 ms; voxel size 0.9 × 0.9 × 5.5 mm); (II) FLAIR (TR/TE 6000/120 ms; voxel size 0.6 × 0.6 × 5.5 mm); (III) DWI (TR/TE 5570/84 ms; voxel size 0.9 × 0.9 × 5.5 mm) |
| 68 | 1.5T Philips. Imaging protocols: (I) T1 (TR/TE 188/2 ms; voxel size 0.9 × 0.9 × 5.5 mm); (II) FLAIR (TR/TE 8500/120 ms; voxel size 0.7 × 0.7 × 5.5 mm); (III) DWI (TR/TE 4176/105 ms; voxel size 1.3 × 1.3 × 5.5 mm) |
| 69 | 3T Philips. Imaging protocols: (I) T1 (TR/TE 288/5 ms; voxel size 0.4 × 0.4 × 5.5 mm); (II) DWI (TR/TE 3857/87 ms; voxel size 1.2 × 1.2 × 5 mm) |
| 70 | 3T Philips. Imaging protocols: (I) T1 (TR/TE 2000/15 ms; voxel size 0.4 × 0.4 × 4 mm); (II) FLAIR (TR/TE 11000/125 ms; voxel size 0.4 × 0.4 × 3.3 mm); (III) DWI (TR/TE 5131/84 ms; voxel size 0.9 × 0.9 × 3.3 mm) |
| 71 | 1.5T Philips. Imaging protocols: (I) FLAIR (TR/TE 11000/140 ms; voxel size 0.5 × 0.5 × 6 mm); (II) DWI (TR/TE 3428/100 ms; voxel size 1 × 1 × 6 mm) |
| 72 | 1.5T Siemens. Imaging protocols: (I) T1 (TR/TE 400/9 ms; voxel size 0.8 × 0.8 × 6 mm); (II) FLAIR (TR/TE 9000/84 ms; voxel size 0.8 × 0.8 × 6 mm); (III) DWI (TR/TE 107463/69 ms; voxel size 1.2 × 1.2 × 6 mm) |
| 73 | 1.5T Siemens. Imaging protocols: (I) T1 (TR/TE 758/14 ms; voxel size 0.7 × 0.7 × 3.3 mm); (II) FLAIR (TR/TE 9000/122 ms; voxel size 0.6 × 0.6 × 6.5 mm); (III) DWI (TR/TE 56802/61 ms; voxel size 1.4 × 1.4 × 6.5 mm) |
| 74 | 1.5T Siemens. Imaging protocols: (I) T1 (TR/TE 2200/3 ms; voxel size 1 × 1 × 1 mm); (II) DWI (TR/TE 160030/68 ms; voxel size 1.2 × 1.2 × 6.5 mm) |
| 75 | 1.5T Siemens. Imaging protocols: (I) DWI (TR/TE 101128/62 ms; voxel size 1.4 × 1.4 × 3.3 mm) |
| 76 | 1.5T Siemens. Imaging protocols: (I) T1 (TR/TE 460/8 ms; voxel size 0.7 × 0.7 × 5.5 mm); (II) FLAIR (TR/TE 9000/87 ms; voxel size 0.7 × 0.7 × 5.5 mm); (III) DWI (TR/TE 126308/75 ms; voxel size 1.2 × 1.2 × 6.5 mm) |
| 77 | 1.5T Siemens. Imaging protocols: (I) T1 (TR/TE 460/8 ms; voxel size 1 × 1 × 1 mm); (II) FLAIR (TR/TE 9000/87 ms; voxel size 0.7 × 0.7 × 5.5 mm); (III) DWI (TR/TE 126308/75 ms; voxel size 1.2 × 1.2 × 6.5 mm) |
| 78 | 1.5T Siemens. Imaging protocols: (I) T1 (TR/TE 10/5 ms; voxel size 1 × 1 × 1 mm); (II) FLAIR (TR/TE 9000/87 ms; voxel size 0.7 × 0.7 × 6.5 mm); (III) DWI (TR/TE 4300/87 ms; voxel size 0.6 × 0.6 × 6.5 mm) |
| 79 | 1.5T Siemens. Imaging protocols: (I) T1 (TR/TE 550/7 ms; voxel size 0.7 × 0.7 × 4.8 mm); (II) FLAIR (TR/TE 9000/84 ms; voxel size 0.7 × 0.7 × 5.8 mm) |
| 80 | 1.5T Siemens. Imaging protocols: (I) FLAIR (TR/TE 9000/84 ms; voxel size 0.7 × 0.7 × 6.5 mm); (II) DWI (TR/TE 93922/60 ms; voxel size 1.4 × 1.4 × 6.5 mm) |
| 81 | 1.5T Siemens. Imaging protocols: (I) T1 (TR/TE 510/9 ms; voxel size 0.5 × 0.5 × 6 mm); (II) FLAIR (TR/TE 11000/106 ms; voxel size 0.5 × 0.5 × 6 mm); (III) DWI (TR/TE 6100/59 ms; voxel size 1.2 × 1.2 × 6 mm) |
